# Assessing the Effectiveness of Different Hyperbaric Oxygen Treatment Methods in Patients with Sudden Sensorineural Hearing Loss

**DOI:** 10.3390/audiolres14020029

**Published:** 2024-03-29

**Authors:** Paweł Rozbicki, Jacek Usowski, Sandra Krzywdzińska, Dariusz Jurkiewicz, Jacek Siewiera

**Affiliations:** 1 Department of Otolaryngology with Division of Cranio-Maxillo-Facial Surgery, Military Institute of Medicine—National Research Institute, 04-141 Warsaw, Poland; 2 Department of Hyperbaric Medicine, Military Institute of Medicine—National Research Institute, 04-141 Warsaw, Poland

**Keywords:** hyperbaric oxygen therapy, sudden sensorineural hearing loss, sudden deafness, audiology

## Abstract

Introduction: Hyperbaric oxygen therapy (HBOT) is one of the treatment methods in patients with sudden sensorineural hearing loss (SSNHL). It is recommended as an elective treatment in patients undergoing steroid therapy. According to current scientific reports, HBOT should be implemented within two weeks after the first symptoms. However, as far as the profile of HBOT is concerned, there are no straightforward recommendations. Methods: The data obtained from the medical records of 218 patients undergoing HBOT for SSNHL at the Military Institute of Medicine—National Research Institute were analyzed statistically for the impact of the duration and the delay in implementing HBOT on the end results of pure-tone audiometry (PTA). Results: A statistically significant hearing improvement in patients undergoing more than 15 cycles of HBOT was detected at all frequencies except for 1500 Hz; in the group reporting for treatment with a delay of more than 10 days, hearing improvement was statistically unsignificant at frequencies of 1500, 3000, and 4000 Hz. Conclusions: The statistical analysis showed that the urgent onset of HBOT could be a significant factor in the therapy of SSNHL.

## 1. Introduction

Hyperbaric oxygen therapy (HBOT) is a treatment method using molecular oxygen in a pressurized environment. Common indications for the therapy are decompression sickness, non-healing of wounds during radiotherapy or diabetes, carbon monoxide poisoning, and brain abscesses [1]. Sudden sensorineural hearing loss (SSNHL) is a disease in which HBOT is applied. This condition causes a significant decline in the performance of hearing organs, which motivates urgent medical consultations at the Emergency Department. In the United States of America, it occurs in 5–27 individuals per 100,000 people annually, and there are approximately 66,000 new cases annually [2,3,4]. Despite numerous studies, the etiology of SSNHL remains unknown. The presumptive underlying causes of SSNHL are autoimmune reactions, traumatic events, metabolic disorders, and vascular abnormalities [5,6,7,8]. Except for the aforementioned reasons, recent studies propose mechanisms including genetic and epigenetic interrelationships, also considering line-1 global DNA methylation, iron homeostasis genes, ferroptosis, and cellular stressors such as iron excess and dysfunctional mitochondrial superoxide dismutase activity [9]. Inadequate treatment or late diagnosis of SSNHL increases the risk of poor recovery. Potential outcomes, such as permanent hearing deficit and tinnitus, significantly decrease the quality of life [10]. Pure-tone audiometry (PTA) remains an essential diagnostic tool for identifying SSNHL, as it involves an analysis of both ways in which the acoustic impulses are conducted. PTA is performed in soundproof rooms with the use of regularly certified devices. A typical setup consists of headphones for air conduction (AC) and bonephones placed on mastoid processes for bone conduction (BC). During the study, clear sounds at specified frequencies are played, with the volume increasing by 5 dB HL until the patient confirms they can hear a tone. This increase occurs by pressing a special button which entails recording of the auditory stimulus threshold on a particular level. Typically, in PTA, the sound in AC is marked for the following frequencies: 125, 250, 500, 1000, 1500, 2000, 4000, 6000, and 8000 Hz. Indications for 6000 and 8000 Hz are not used in BC due to an intensified muffling of these sounds by cranial bones. This enables differentiating sensorineural, conductional, and mixed hearing losses. Sensorineural hypoacusis involves lowering both the air and bone audiometric curves.

Sensorineural hypoacusis implies damage to the acoustic organ above the conducting apparatus, that is, the organ of Corti and auditory pathway neurons along the cochlear nerve, cochlear nuclei, lateral lemniscus, medial geniculate body, and other central nervous system structures reaching the auditory cortex. A detailed distinction of the etiologies of various types of sensorineural hypoacusis was made during audiological studies, such as brainstem auditory evoked potential measurements, otoacoustic emission, verbal audiometry, and electrocochleography. The diagnostic criterion of SSNHL is an increase in the hearing threshold stimulus to >30 dB HL over at least three audiometric frequencies, developing over no longer than 72 h [11]. In compliance with the recommendations of the Academy of Otolaryngology–Head and Neck Surgery (AAO-HNS)’s Clinical Practice Guidelines: Sudden Hearing Loss, the primary treatment methods of SSNHL are oral, intravenous, or intratympanic steroid therapies (STs). Each method has advantages and disadvantages, so a choice of a given method should be made case by case. The AAO-HNS recommends a standard dose of systemic ST of 1 mg/kg of body mass for prednisone, with a maximum dose of 60 mg. Nonetheless, a detailed schedule of systemic ST application in SSNHL remains debatable and is the subject of scientific research. The AAO-HNS, in its Clinical Practice Guidelines: Sudden Hearing Loss, recommends HBOT within 14 days after the first symptoms as an elective treatment. HBOT might be used to improve outcomes in patients with SSNHL in conjunction with steroids or as salvage therapy [12,13]. Other relevant medical societies and associations also recommend HBOT [14,15,16]. HBOT can be distinguished into initial treatment or salvage treatment. Treatment combined with steroid therapy within two weeks of symptom onset is known as initial treatment. Salvage treatment means HBOT combined with steroid therapy implemented within one month after the onset of SSNHL. This involves exposing the patient to 100% oxygen at a pressure of 1.5–2 ATA; the purposes of an increased supply of oxygen to the cochlea are a positive impact on the patient’s immunity and hemodynamics, a reduction in hypoxia and edema, and potentiation of normal host responses to infection and ischemia [17]. The healing impact of HBOT may be related to increased red blood cells, hematocrit, hemoglobin concentration, and superoxide dismutase [18]. Moreover, HBOT preserves the microcirculation by reducing venular leukocyte adherence and inhibiting progressive adjacent arteriolar vasoconstriction [17]. Currently, no guidelines point to a particular HBOT protocol in patients with SSNHL, but a greater interest in this area has recently been observed [19]. The cited study demonstrated a dependence of pressure applied in the hyperbaric chamber on hearing improvement. It appears that patients applied with a pressure of 2.5 ATA had better treatment results than those treated with 1.5 ATA. In the examined group, there were no statistically significant differences in the decrease in hearing threshold stimulus with respect to the total time of compression (1 vs. 2 h). A selective influence of pressure values on hearing threshold stimulus was, however, demonstrated by Krajcovicova et al. [20]. Application of 2.0 ATA improved treatment results in the speech frequency spectrum. Compressions of 2.5 ATA proved more effective at lower frequencies. Further study showed that a pressure of 2.0 ATA is ineffective in the treatment of hearing loss encompassing octave bands of 1–2 kHz. Brief instructions available in the present-day scientific literature are not backed by research on the recommended number of cycles of hyperbaric oxygen therapy. For instance, LeGros et al. [21] mention 10–20 days of therapy. HBOT protocols applied until now result from experiences at research centers and the protocols applied in other diseases treated with HBOT. The development of recommendations requires a statistical analysis of treatment results in patients suffering from SSNHL subjected to different protocols, constituting this manuscript’s foundation. A significant proportion of patients with SSNHL do not fully recover despite proven idiopathic disease remissions. The risk of permanent hearing deficits and an impairment stemming from them is a reason behind scientific research on therapy optimization. Such research aims to maximize the percentage of full recovery in patients and to reduce adverse reactions to the performed interventions. This study aimed to evaluate the influence of different HBOT profiles on hearing improvement in patients with SSNHL.

## 2. Materials and Methods

The medical records of patients admitted to the Department of Hyperbaric Medicine, Military Institute of Medicine—National Research Institute, and the Department of Otolaryngology with Division of Cranio-Maxillo-Facial Surgery, Military Institute of Medicine—National Research Institute due to SSNHL between January 2018 and December 2019 were analyzed retrospectively. The number of patients treated by the Institute during the indicated period determined the scale of the study. The eligibility criteria for the research included diagnosed SSNHL and completeness of medical records, including details on whether the patient had PTA before and after HBOT. A diagnostic criterion of SSNHL, according to the guidelines of the AAO-HNS, is an increase in the hearing threshold stimulus to >30 dB HL over at least three audiometric frequencies, developing over no longer than 72 h.

Exclusion criteria were as follows: age < 18 years, age > 85 years, HBOT initiated 30 days after the first symptoms of SSNHL, coexistent cerebrospinal inflammation, neuropsychiatric disorders, Ménière’s disease, hereditary hearing disorders, inner ear malformations, facial nerve neuromas, acoustic neuromas (or other tumors of the inner auditory canal, cerebellopontine angle), bilateral SSNHL, subsequent episode of SSNHL, and air–bone gaps on PTA.

PTA results were the key variables considered for the SSNHL diagnosis. The tests were conducted using an Interacoustics AC40 audiometer for the following frequencies [Hz] for air conduction (AC) (125, 250, 500, 1000, 1500, 2000, 3000, 4000, 6000, 8000) and bone conduction (BC) (125, 250, 500, 1000, 1500, 2000, 3000, 4000). To prevent the non-test ear from influencing the results, the study used tone masking. A statistical analysis was conducted only for BC, given the nature of SSNHL. Since PTA is subjective, its result as the only variable was potentially biased. Patients with diagnosed SSNHL were treated with ST schemes. Details on the pharmacotherapy used can be found in our team’s previous work [22]. Patients underwent HBOT with the following protocol: compression to 2.5 ATA, total compression/decompression time: 10 min (0.025 m/s), oxygenation 3 × 20 min with 100% as a breathing factor, and oxygen breaks 2 × 5 min performed routinely to prevent toxic effect on the lungs. A medical staff member (a nurse, a doctor, or a paramedic) was present in the chamber during HBOT. The HBOT cycles were conducted daily, once a day. Compression during hyperbaric therapy was performed with a BAROXHBO device. Most patients underwent 15 cycles (1 cycle being equivalent to 1 day) of HBOT, given the previous experience of the institute. The patients were divided into subgroups for statistical analysis according to the delay in implementing HBOT (<5 days, 5–10 days, >10 days) and the number of cycles/duration of HBOT (<15 cycles, 15 cycles, >15 cycles). After the start of HBOT, patients underwent a monitoring hearing test. The data concerning the above-mentioned variables were extracted from the medical records of patients treated in the Department of Hyperbaric Medicine and Department of Otolaryngology with the Division of Cranio-Maxillo-Facial Surgery, Military Institute of Medicine—National Research Institute.

### Statistical Analysis

The results were tabulated. First, the mean hearing threshold in patients before and after treatment was summarized for the entire study group. Using the visual method and the Kolmogorov–Smirnov test, a normal distribution was found in the studied variables. Next, using a paired Student’s *t*-test, the significance of differences between the means before and after the treatment was verified.

Subsequently, the relationship between the delay in hyperbaric therapy and the end results of treatment was analyzed. After confirming a normal distribution in the subgroups, statistical analysis was performed using a paired Student’s *t*-test. The first step was to compare the significance of differences in the mean hearing threshold in PTA before and after the treatment across the subgroups.

The final step of the statistical analysis was to compare the statistical significance of hearing improvement according to the number of hyperbaric chamber cycles. After confirming the normal distribution of the variables, the data were analyzed using a paired Student’s *t*-test.

## 3. Results

The statistical analysis included 218 patients (mean age: 48.8 ± 14.5 years; 117 males, 101 females). Patients not meeting the study criteria were excluded from the study and the analysis. Each patient was observed for at least two months after admission to the Military Institute of Medicine—National Research Institute. No patient was disqualified due to HBOT contraindications. Patients administered <15 cycles of HBOT terminated treatment for personal reasons that were not related to the treatment itself.

The studied patients demonstrated a typical distribution for age structure and hearing depth in the Kolmogorov–Smirnov test. The average patient delay in reporting to the Department of Hyperbaric Medicine—Military Institute of Medicine—National Research Institute after the onset of SSNHL symptoms was 8.2 (±6.6) days. The average hearing threshold stimulus in the study group before the treatment was 54.6 (±31.3) dB HL and it was 39.6 (±32.2) dB HL after the treatment.

A significant lowering of hearing threshold was observed in BC, which directly indicates a recovery from SSNHL. The results are presented in Table 1.

Then, the respective results of hyperbaric treatment were analyzed. In the first step, patients treated with HBOT were divided into subgroups according to the delay in therapy initiation: <5 days (75 patients), 5–10 days (95 patients), and >10 days (48 patients). In the next step, the group was divided according to the number of applied compressions: <15 cycles (23 persons), 15 cycles (176 persons), and >15 cycles (19 persons). A normal distribution in variables resulting from the PTA study was confirmed for all the groups. This enabled a parametric analysis during further evaluation of the groups. The results of the Student’s *t*-test with respect to the delay in HBOT are shown in Table 2.

The analysis presented here shows statistically significant improvements across all frequencies in the patient groups studied except for 1500, 3000 Hz, and 4000 Hz in the group who reported for treatment with a delay of more than 10 days.

Next, the groups were divided by therapy duration (number of cycles) and analyzed using a Student’s *t*-test (Table 3).

A statistically significant hearing improvement was demonstrated for patients treated with 15 cycles or less across all frequencies analyzed. In patients treated with more than 15 cycles, hearing improvement was statistically significant across all frequencies except for 1500 Hz.

## 4. Discussion

The statistical analysis results complied with the purpose of the study, which was to confirm the impact of the HBOT profile on treatment outcomes. In the groups compared, delaying HBOT more than 10 days from the time of the first SSNHL symptoms was associated with a reduced treatment efficacy at 1500, 3000, and 4000 Hz. This is in line with current scientific evidence [12,20,23,24,25]. Cavaliere et al. demonstrated a complex relationship between the choice of therapeutical strategy for SSNHL and hearing improvement [26]. The cited report compared the HBOT results with and without simultaneous application of ST. The treatment was particularly effective 14 days after the first symptoms. Moreover, the use of ST with HBOT resulted in less satisfying therapy results in patients admitted for treatment up to seven days after the first symptoms of SSNHL. There is a need for further research in this field, focusing on the relationship between oxidative stress [18] occurring at the early stage of SSNHL and the choice of treatment protocol. The comparison of HBOT delays indicated that in patients admitted to hospital after 10 days, the hearing improvement was statistically significant for a frequency of 250, 500, 1000, and 2000 Hz. This means that hearing improvements in the examined group of patients were not statistically significant at higher frequencies in the speech spectrum. Tae-Min Rhee’s team [27] conducted a meta-analysis encompassing 16 unrandomized tests and three RCTs, which showed the statistically significant benefits of HBOT lasting longer than 1200 min. Their findings are in agreement with our study. HBOT requires regular visits to a medical facility at a fixed time. These facilities may be located a certain distance from the patient’s residence, causing difficulties related to commuting and accommodation. For this reason, one may expect differences in the availability of HBOT for patients with SSNHL depending on the country, and the distribution of hyperbaric medicine centers in the region inhabited by patients may impact the treatment outcomes. One potential solution to such problems would be the development of new HBOT facilities. However, this may prove problematic, particularly in countries with low spending on healthcare. Patients may have also resigned from the therapy due to a lack of motivation for regular visits to their medical facility. A patient undergoing therapy should, thus, always be informed by an attending physician on the chances and risks of the therapy. The communication between a doctor and a patient should be considered a key determinant of end-treatment results. In their meta-analysis encompassing 106 correlational studies and 21 experimental interventions, Zolnierek et al. demonstrated that the communication skills of medical staff strongly correlate with patients following their guidelines. The recommendations offered by staff members trained in soft skills were followed 1.62 times more often than by doctors who did not possess such skills [28].

Our study results also revealed a decreased significance of hearing improvements in patients treated with more than 15 cycles of HBOT. This could be induced by the negative impact of oxidate stress caused by hyperbaric oxygen or the smaller number of enrolled patients in this group. These relations should be solved in further studies.

The experience of the center and the retrospective character of this study directly led to the uneven distribution of patients in groups divided by the number of treatment cycles in a hyperbaric chamber. Differences in the number of patients in the study groups did not cause deviations from the normal distribution in the groups, which enabled their comparison using parametric statistical tests. The limitation of this study was the lack of a control group of patients who did not undergo HBOT. This was associated with clinical practice in our institute, based on evidence-based medicine describing the positive influence of HBOT on SSNHL [12,13,14,15].

Studies opposing the recommendations of the AAO-HNS’s Clinical Practice Guideline: Sudden Hearing Loss [11] report no significant differences in end treatment results between groups treated exclusively with ST as opposed to groups treated with ST and HBOT [29]. In the cited study, there are the following differences in the HBOT protocols: intervals between compressions—5 min vs. 10 min—and minor differences in the decompression time—10 min vs. 6–12 min. In comparison with Skarżyński et al., the hearing improvement in our study was 15.3 dB HL vs. 14.1 dB HL. It can be expected that these dependencies will not achieve statistical significance. A difference in subjective hearing may also be clinically irrelevant. Nevertheless, a statistical calculation of raw data from both teams would need to be conducted to exclude the influence of a specific HBOT profile.

A metanalysis conducted by Eryigit et al. [30] of 16 articles published between 2002 and 2018 proved the relationship between the depth of hearing impairments and the final outcomes of HBOT treatment in SSNHL. In the cited study, no significant difference was demonstrated between the HBOT and control groups. On the contrary, in patients with severe and profound SSNHL, the hearing improvement was statistically significant. In the group of patients enrolled in our study, a significant number of patients presented moderate hearing impairments that could reduce the salience of the results. Studying the relationship between the severity of SSNHL and the influence of HBOT on final outcomes requires further prospective studies in diverse HBOT protocols.

Opposing to study conducted by Eryigit [30], Choi et al. stated there were no differences in hearing improvement between the HBOT and control group of patients with severe to profound SSNHL [31]. However, the differences of hearing improvement rate were observed in group of patients with diabetes mellitus. The cited study was performed on a group of patients undergoing an HBOT protocol including ventilation for 90 min per session with 2,4 ATA pressure. The number of sessions administered for patients was described as over 14 sessions. This means that the hyperbaric session properties were comparable to our study. On the other hand, the delay to HBOT was higher in our group than for the subjects enrolled by Choi et al. This could be a reason why hearing gains are higher in Choi’s study in comparison to our 53.3 dB HL (53.4% difference prior to initial PTA) and 39.1 dB HL (39.6% difference prior to initial PTA) vs. 15,0 dB (27.5% difference prior to initial PTA). This corresponds with results of our study, where the hearing improvement was more significant in the group of patients with minor delays to HBOT administration.

Dynamically developing prosthetics are often essential in the treatment of hypoacusis, especially considering the poor prognosis for full recovery in many cases of SSNHL. Promising results of surveys on improvements in quality of life and quality of hearing were observed in a multicenter prospective examination of life quality in patients with surgically implanted cochlear implants (the Health Utility Index increased by 33%, SSQ improvement in the quality of comprehending speech of 180%, spatial hearing increased by 135%, quality of hearing increased by 98%) [32]. The priority remains to achieve the best possible results in improving hearing, despite the development of medical technologies that improve hypoacusis. Current scientific reports lack research that would standardize an HBOT profile used in the treatment of SSNHL regarding the number of compressions and the duration of a single compression cycle. The results of our study encourage further research across a wider population to establish an optimal therapy scheme, which may lead to an improvement in the SSNHL treatment outcomes.

## 5. Conclusions

In the group of patients who received HBOT after 10 days, there was no statistically significant improvement in the hearing threshold in high-frequency PTA. Thus, it seems beneficial to start HBOT for SSNHL within the first 10 days of illness. The statistical significance of hearing improvements decreased in the group of patients treated with more than 15 cycles of HBOT. Further prospective studies with standardized and randomized therapeutic protocols are required.

## Figures and Tables

**Table 1:** BC before and after combined treatment with ST and HBOT.

Frequency [Hz]	Before [dB]	After [dB]	Difference	*p*
250	41.22	26.03	15.19	<0.001
500	48.05	28.68	19.37	<0.001
1000	49.05	30.52	18.52	<0.001
1500	69.40	32.72	36.68	<0.001
2000	52.49	34.86	17.63	<0.001
3000	56.76	38.11	18.65	<0.001
4000	54.03	38.67	15.35	<0.001

**Table 2:** Delay in HBOT and improvement in hearing at different frequencies.

Frequency [Hz]	<5 Days (*p*-Value; Difference [dB])	5–10 Days (*p*-Value; Difference [dB])	>10 Days (*p*-Value; Difference [dB])
250	*p* < 0.001; 17.6	*p* < 0.001; 14.5	*p* < 0.05; 11.4
500	*p* < 0.001; 21.6	*p* < 0.001; 17.9	*p* < 0.001; 18.2
1000	*p* < 0.001; 21.3	*p* < 0.001; 17.7	*p* < 0.05; 13.5
1500	*p* < 0.001; 24.2	*p* < 0.001; 21.8	*p* > 0.05; 8.2
2000	*p* < 0.001; 19.8	*p* < 0.001; 16.2	*p* < 0.05; 12.1
3000	*p* < 0.001; 18.2	*p* < 0.001; 15.9	*p* > 0.05; 10.2
4000	*p* < 0.001; 15.9	*p* < 0.001; 13.7	*p* > 0.05; 10.9

**Table 3:** Cycle count in HBOT and improvement in hearing at different frequencies.

Frequency [Hz]	<15 Cycles (*p*-Value; Difference [dB])	15 Cycles (*p*-Value; Difference [dB])	>15 Cycles (*p*-Value; Difference [dB])
250	*p* < 0.001; 14.3	*p* < 0.001; 14.9	*p* < 0.05; 24.4
500	*p* < 0.001; 18.8	*p* < 0.001; 19.4	*p* < 0.05; 28.1
1000	*p* < 0.001; 17.7	*p* < 0.001; 18.2	*p* < 0.01; 24.7
1500	*p* < 0.001; 19.6	*p* < 0.001; 20.7	*p* > 0.05; 33.6
2000	*p* < 0.001; 16.0	*p* < 0.001; 15.7	*p* < 0.05; 25.3
3000	*p* < 0.001; 15.0	*p* < 0.001; 14.9	*p* < 0.05; 26.0
4000	*p* < 0.001; 12.6	*p* < 0.001; 11.8	*p* < 0.01; 29.7

## Data Availability

Medical data are available at the Military Institute of Medicine—National Research Institute.
